# Supplementary material for: MEmilio: a high performance Modular EpideMIcs simuLatIOn software for multi-scale and comparative simulations of infectious disease dynamics
Source: Sci Rep. 2026 Aug 27;16:26950. doi: 10.1038/s41598-026-66481-6 (PMC13522579; doi:10.1038/s41598-026-66481-6)
Supplement: Supplementary file 1 [file 41598_2026_66481_MOESM1_ESM.pdf]

Supplementary Information for: MEmilio: A high performance Modular EpideMIcs simuLatIOn software for multi-scale and comparative simulations of infectious disease dynamics

Contents

A List of abbreviations **2**

B Mathematical tools and technical functionality **2**
- B.1 Numerical solvers and technical details . . . 2
  - B.1.1 Numerical solvers for ODE-based models . . . 3
  - B.1.2 Numerical solvers for IDE-based models . . . 3
  - B.1.3 Numerical solvers for SDE-based models . . . 4
- B.2 Parallelization with shared and distributed memory . . . 4
- B.3 Optimal control through direct single shooting . . . 5

C General calibration processes **5**
- C.1 Calibration with simulation-based inference methods . . . 5
- C.2 Calibration with particle filters . . . 5

D Details on models and calibration for influenza, Ebola, and spatio-temporal SARS-CoV-2 use cases **6**
- D.1 Calibration with particle filters for influenza and Ebola . . . 6
  - D.1.1 SDE-SIRS model for influenza . . . 6
  - D.1.2 ODE-SEIRDB model for Ebola . . . 6
- D.2 Calibration with BayesFlow for spatio-temporal SARS-CoV-2 . . . 7

E Models and parameters for scenario analysis of NPIs **9**

F Models and parameters for model comparison **9**
- F.1 Temporal-hybrid model comparison and scaling . . . 9
- F.2 Comparison of ABM and ODE-, LCT- and IDE-based models . . . 10

G Cross-implementation performance: C++, Python, and R **15**
- G.1 Model specification and metapopulation extension . . . 15
- G.2 Implementations . . . 15
- G.3 Simulation parameters . . . 15

H Benchmarking Graph Neural Network surrogates against metapopulation model **16**
- H.1 Surrogate model architecture and training . . . 16
- H.2 Benchmark setup . . . 16

I Performance evaluation of PBMs and MPMs **17**
- I.1 Scaling with respect to regions in ODE-, LCT-, and IDE-based MPM . . . 17
- I.2 Strong scaling with respect to the number of simulations . . . 17

J Performance evaluation of the mobility-based ABM **19**
- J.1 Comparisons with other frameworks . . . 19
- J.2 Strong scaling with respect to the number of simulations . . . 19

- The MEmilio software framework with more than 70 code examples is available at: https://github.com/SciCompMod/memilio
- A comprehensive list of tutorials and exercises for the MEmilio software framework is available at: https://github.com/SciCompMod/memilio-tutorials/
- MEmilio's documentation with examples and explanations is available at: https://memilio.readthedocs.io/en/latest/
- MEmilio's simulations (as used in this and other selected manuscripts) are available at: https://github.com/SciCompMod/memilio-simulations

A List of abbreviations

In this section, we provide a list of abbreviations as used in the manuscript and the supplementary information.

- MEmilio: a high performance Modular EpideMIcs simuLatIOn software
- ODE: ordinary differential equation
- LCT: Linear Chain Trick
- SDE: stochastic differential equation
- IDE: integro-differential equation
- PBM: population-based model
- MPM: metapopulation model
- ABM: agent-based model
- dABM: diffusive agent-based model
- GNN: graph neural network
- RSV: respiratory syncytial virus
- COVID-19: coronavirus disease 2019
- SARS-CoV-2: severe acute respiratory syndrome coronavirus 2
- ICU: intensive care unit
- NPIs: nonpharmaceutical interventions
- SBI: simulation-based inference
- ABC: approximate Bayesian computation
- SMC: sequential Monte Carlo
- MCMC: Markov chain Monte Carlo
- PMCMC: particle Markov chain Monte Carlo
- PF: particle filter
- MH: Metropolis-Hastings
- PMMH: pseudo-marginal Metropolis-Hastings
- CI: credibility interval
- AD: automatic differentiation
- IPOPT: Interior Point OPTimizer
- MPI: message passing interface

B Mathematical tools and technical functionality

In this section, we first give a brief description of the mathematical tools and technical functionality that are available with MEmilio. Precise usage as applied in this manuscript is described in Sections D–I and in the simulation code published open-source.

B.1 Numerical solvers and technical details

All models in MEmilio are complemented by a `Simulation` class. MEmilio provides a standard implementation for ODE-based `CompartmentalModels` and `FlowModels` as well as a `GraphSimulation` that allows coupling multiple local simulations. More involved models, like the SDE-, IDE-, and agent-based models, define their own `Simulation`. All simulations have an `advance()` function that runs the simulation up to a user-defined time point, and in the case of the ABM also allows adding `Loggers` to specify which information to extract. The PBMs collect their results in a `TimeSeries`, storing the simulated `Population` by compartments over time, accessible through a `get_result()` function.

B.1.1 Numerical solvers for ODE-based models

All ODE-based models use the same scheme. `Simulation::advance()` defines a function denoted `DerivFunction`, that expects a time t and population $y(t)$ and returns the right-hand side of the model $f(y(t),t) = \frac{\mathrm{d}y(t)}{\mathrm{d}t}$, passing it to the numerical integrator.

The integrator (`OdeIntegrator`) is model independent; it only assumes that $\frac{\mathrm{d}y}{\mathrm{d}t}$ and y have the same shape. It manages the result `TimeSeries`, adding new entries as it integrates from a given start time to a final time. The integrator supports explicit integration schemes with both fixed and adaptive time stepping, and the scheme can be set from the `Simulation`. By default, we use the Boost implementation [1] of the Cash-Karp 54 Runge-Kutta method [2]. The `DerivFunction` is used to evaluate derivatives at time points determined by the integration scheme to iteratively compute the population at the next time step.

B.1.2 Numerical solvers for IDE-based models

The implemented IDE-based model is solved using a nonstandard finite difference scheme that was proposed by Messina et al. [3] and that was extended to a SECIR-type model with flow formulation Wendler et al. [4]. Here, we give a brief overview of the numerical scheme for an SIR model defined by

$$\begin{aligned}
\frac{\mathrm{d}S}{\mathrm{d}t} &= -\sigma_{S,I},\\
\frac{\mathrm{d}I}{dt} &= \sigma_{S,I} - \sigma_{I,R},\\
\frac{\mathrm{d}R}{dt} &= \sigma_{I,R},
\end{aligned}$$

where the flows between the infection states are given by

$$\begin{aligned}
\sigma_{S,I} &= S(t)\lambda(t),\\
\sigma_{I,R}(t) &= -\int_{-\infty}^{t} {\gamma_I}'(t-x)\,\sigma_{S,I}(x)\,\mathrm{d}x,
\end{aligned}$$

and the force of infection is defined via

$$\lambda(t) = \frac{\phi(t)\rho(t)}{N}\int_{-\infty}^{t} \xi(t-x)\,\gamma_I(t-x)\,\sigma_{S,I}(x)\,\mathrm{d}x.$$

ϕ is the average number of daily contacts, ρ is the transmission probability on contact and ξ denotes the fraction of infected individuals that are not isolated. $\gamma_I(\tau)$ determines the expected proportion of individuals that are still infected τ days after transmission.

Let $\Delta t > 0$ be the fixed step size and $t_i = i\Delta t$, $i \in \mathbb{Z}$, equidistant time points. In the following, we use the notation $\widehat{x}$ for the respective discretized version of the variable x. The number of Susceptibles is determined by the number of Susceptibles in the previous time step as well as the force of infection from the previous time step by

$$\widehat{S}(t_{i+1}) = \frac{\widehat{S}(t_i)}{1+\Delta t\widehat{\lambda}(t_i)}.$$

Using the relation $\frac{\mathrm{d}S}{\mathrm{d}t} = -\sigma_{S,I}$, we obtain

$$\widehat{\sigma}_{S,I}(t_{i+1}) = \widehat{S}(t_{i+1})\widehat{\lambda}(t_i),$$

which is a right endpoint approximation in $\widehat{S}$ and a left endpoint approximation in $\widehat{\lambda}$ where $\widehat{\lambda}(t_i)$ is known from the previous time step.

Based on this, we compute the subsequent flow by

$$\widehat{\sigma}_{I,R}(t_{i+1}) = -\Delta t\sum_{j=a}^{i}\widehat{\gamma}_I'(t_{i+1-j})\widehat{\sigma}_{S,I}(t_{j+1}),$$

where $\widehat{\gamma}_I'$ is a suitable approximation of γ_I'. The remaining compartments are computed via

$$\widehat{I}(t_{i+1}) = \widehat{I}(t_i) + \Delta t\left(\widehat{\sigma}_{S,I}(t_{i+1}) - \widehat{\sigma}_{I,R}(t_{i+1})\right),$$

$$\widehat{R}(t_{i+1}) = \widehat{R}(t_i) + \Delta t\, \widehat{\sigma}_{I,R}(t_{i+1}).$$

Finally, we compute the force of infection by

$$\widehat{\lambda}(t_{i+1}) = \frac{\phi(t_{i+1})\rho(t_{i+1})}{N} \Delta t \sum_{j=a}^{i} \xi(t_{i+1-j})\, \gamma_I(t_{i+1-j})\, \widehat{\sigma}_{I,R}(t_{j+1}),$$

where we approximate the integral by a rectangular rule with a right endpoint approximation in $\widehat{\sigma}_{I,R}$ and a left endpoint approximation in ξ and γ_I. It can be shown that this discretization scheme conserves essential biological properties of the continuous model [3, 4]. Current developments cover the analysis and adaptation of higher-order solvers [5], in particular making these numerical schemes also available for systems with contact change points [6].

B.1.3 Numerical solvers for SDE-based models

For the numeric approximation of SDEs, MEmilio utilizes the structural similarity between the Euler approximation scheme for ODEs and the Euler-Maruyama approximation scheme for SDEs.

Consider the SDE

$$\mathrm{d}X(t) = a(X,t)\mathrm{d}t + b(X,t)\mathrm{d}W,$$

with the initial condition $X(0) = x_0$, where W denotes a standard Wiener process. For a fixed step size $\Delta t > 0$, the Euler-Maruyama scheme approximates the solution on the interval $I = [0, T]$ as follows.

We partition I into discretization points

$$0 = \tau_0 < \tau_1 < \ldots < \tau_N \leq T,$$

with $\tau_{i+1} - \tau_i = \Delta t$ for all $i = 0, \ldots, N-1$.

The recursive approximation is given by

$$\begin{aligned} Y_0 &= x_0, \\ Y_{i+1} &= Y_i + a(Y_i, \tau_i)\Delta t + b(Y_i, \tau_i)\Delta W_i, \end{aligned}$$

with $\Delta W_i = W(\tau_{i+1}) - W(\tau_i)$, where the ΔW_i are independent and identically distributed normal random variables with mean zero and variance Δt. Using that $\sqrt{\Delta t} N(0,1) \sim N(0, \Delta t)$, we can rewrite the above scheme as follows

$$\begin{aligned} Y_0 &= X_0, \\ Y_{i+1} &= Y_i + a(Y_i, \tau_i)\Delta t + \frac{b(Y_i, \tau_i)}{\sqrt{\Delta t}} \Delta \overline{W}_i \Delta t, \end{aligned}$$

with $\Delta \overline{W}_i \sim N(0,1)$. With this formulation, MEmilio uses the Euler solver to solve the SDE.

B.2 Parallelization with shared and distributed memory

Within different modules and kernel functionality, MEmilio uses different approaches of parallelization.

For most PBMs and MPMs, MEmilio provides a sequential and a parallelized ensemble-run functionality, where the latter executes a user-defined number of runs with a specified number of MPI ranks [7]. At the end of the simulation, the first rank collects all simulation outputs and orders them to compute the 5th, 25th, median, 75th and 95th percentiles such that credible intervals of 50 % and 90 % are returned by default. A shared memory parallelism for MPMs, using OpenMP [8] to compute different graph nodes and mobility in parallel, has been used in various occasions on development branches. However, since generally, a lot of parallel simulations have to be conducted, the parallelization across different model runs is more efficient and, thus, preferred by default.

While we also provide MPI-parallelized ensemble-run functionality with the computation of summary statistics for the ABM, we furthermore use shared memory parallelism through OpenMP [8] inside its heavier computation kernels. Since, at each time step, agents only interact within the same location, we allow computing interactions and movement simultaneously for each location.

B.3 Optimal control through direct single shooting

In the context of epidemiological models, optimal control [9] provides a framework for determining optimal intervention strategies [10, 11]. In MEmilio, optimal control problems are formulated using a direct single shooting approach, which relies on control discretization and state integration, also referred to as the sequential method in the literature [12]. The nonlinear optimization problem is solved using the interior-point solver IPOPT [13].

To enable optimal control, MEmilio integrates automatic differentiation (AD) as a core component. AD allows the computation of exact derivatives of model outputs with respect to the control variables. The AD implementation in MEmilio builds on code developed by the group Software and Tools for Computational Engineering (STCE) at RWTH Aachen University [14] and supports both forward- and reverse-mode automatic differentiation through operator overloading. In this approach, standard floating-point types used in computations are substituted with an active AD type. Arithmetic operators and relevant MEmilio components are overloaded for the active type such that derivative information is automatically propagated alongside the stored variable. MEmilio provides AD for a wide range of epidemiological ODE-based models, ranging from compartmental models to more intricate graph-based formulations.

C General calibration processes

In this section, we briefly describe MEmilio's general functionality of being coupled with external tools for simulation-based inference (SBI) and particle filters (PF). Precise usage for this manuscript is described in Section D.

C.1 Calibration with simulation-based inference methods

MEmilio supports parameter inference via SBI, which is particularly well-suited for mechanistic epidemiological models where simulations can be conducted efficiently while the likelihood function may be expensive to evaluate, difficult to specify, or just analytically unavailable due to complex stochastics. In SBI, inference is performed by combining a parameter prior with simulations generated from the model, replacing explicit likelihood evaluations by learned or approximate inference procedures [15].

A simulator f takes a parameter vector $\theta \in \Theta$ and produces model outputs $y = f(\theta)$, typically time series such as compartment trajectories, incidences, or derived observables. Given a prior distribution $p(\theta)$, SBI proceeds by repeatedly drawing parameters from the prior, simulating corresponding trajectories, and using the resulting pairs (θ, y) to infer the posterior distribution $p(\theta \mid y_{\text{obs}})$ for an observed dataset y_{obs}.

This modular structure separates the model from the inference engine: MEmilio provides the simulator output y, while inference is carried out by external libraries (e.g., BayesFlow [16] and pyABC [17]) which need simulated data and prior samples to approximate the posterior distribution. While MEmilio's backend can be used for offline generation of training data for neural network-based inference, the Python interface of MEmilio is designed such that its simulator can be coupled online into SBI libraries. The same simulator can therefore be used with multiple inference strategies: (i) neural posterior estimation (as implemented in BayesFlow), and (ii) approximate Bayesian computation with sequential Monte Carlo (ABC-SMC as implemented in pyABC). While the main manuscript focuses on the BayesFlow integration, the overall structure applies analogously to pyABC: in both cases, inference relies on repeated calls to the simulator and a prior specification over model parameters.

C.2 Calibration with particle filters

Particle Markov chain Monte Carlo (PMCMC) methods combine a PF with a Markov chain Monte Carlo (MCMC) scheme to enable Bayesian inference for nonlinear and non-Gaussian state space models, where the likelihood is intractable. The main idea is that a PF can produce an unbiased estimate of the likelihood and this estimate can then be used inside an MCMC acceptance step leaving the target density, i.e., the parameter posterior, invariant [18].

A PF is a sequential Monte Carlo (SMC) technique based on importance sampling with resampling primarily used for estimating the latent state distribution by an ensemble of simulated trajectories (particles) [19]. The main idea is to iterate over the following three steps (i) propagate forward through the latent dynamics, (ii) weight according to agreement with the data under the observation model, and (iii) resample to discard low-weight particles and focus computational effort on particles that are more consistent with the data. Importantly for parameter inference, a PF produces as a by-product a likelihood estimate obtained by accumulating the normalized weights of each observation step.

In this work, we leverage a so-called bootstrap filter, where the propagation step is performed by simulating directly from the model's latent dynamics rather than using a tuned proposal. The trade-off is that for informative observations or a high-dimensional model, the weights can become highly uneven and a large number of particles must be used to avoid particle degeneracy. We then further combine this with an outer Metropolis-Hastings (MH) scheme by replacing the likelihood term in the acceptance step with the PF estimate leading to a version of a pseudo-marginal Metropolis-Hastings (PMMH) algorithm [20]. The use of a bootstrap filter within an MH scheme yields a practically useful and easily applicable version of simulation-based exact Bayesian inference for stochastic models [21].

D Details on models and calibration for influenza, Ebola, and spatio-temporal SARS-CoV-2 use cases

In this section, we provide details on models and calibration as used for Fig. 4 in the main manuscript.

D.1 Calibration with particle filters for influenza and Ebola

D.1.1 SDE-SIRS model for influenza

We use an SIRS model with seasonal transmission as used for influenza in Edlund et al. [22], using an SDE instead of an ODE formulation. The model equations are given by

$$\begin{aligned}
\mathrm{d}S &= -\beta(t)\frac{S\,I}{N}\,\mathrm{d}t + \frac{1}{T_R}R\,\mathrm{d}t - \sqrt{\beta(t)\frac{S\,I}{N}}\,\mathrm{d}W_1 + \sqrt{\frac{1}{T_R}R}\,\mathrm{d}W_3,\\
\mathrm{d}I &= \beta(t)\frac{S\,I}{N}\,\mathrm{d}t - \frac{1}{T_I}I\,\mathrm{d}t + \sqrt{\beta(t)\frac{S\,I}{N}}\,\mathrm{d}W_1 - \sqrt{\frac{1}{T_I}I}\,\mathrm{d}W_2,\\
\mathrm{d}R &= \frac{1}{T_I}I\,\mathrm{d}t - \frac{1}{T_R}R\,\mathrm{d}t + \sqrt{\frac{1}{T_I}I}\,\mathrm{d}W_2 - \sqrt{\frac{1}{T_R}R}\,\mathrm{d}W_3.
\end{aligned}$$

The effective transmission rate $\beta(t)$ is given by

$$\beta(t) = \beta_0\left[\sum_i\left(\kappa_i + (1-\kappa_i)\frac{g(t-t_i,\sigma_i)}{g(0,\sigma_i)}\zeta(t,t_i)\right)\right],$$

where β_0 is the baseline transmission coefficient, $(1-\kappa_i)$ is the fraction by which the transmission varies during season i, t_i is the day of the peak transmission for season i, $\zeta(t,t_i)$ joins seasons with an apodization window of ± 30 days and σ_i is the standard deviation of the Gaussian probability distribution function $g(x,\sigma_i)$.

The model is calibrated to weekly incidences of influenza in Germany, as provided by the Robert-Koch Institute [23]. Summed over all age groups, we extracted the three seasons of the pre-COVID-19 period 2016-W32 (Start day: August 7th, 2016) to 2019-W31 (End day: August 5th, 2019).

As observables, we used the I compartment with additive Gaussian noise, where the variance is scaled by the size of the I compartment with

$$Y \sim \mathcal{N}(I, \sigma I).$$

The initial state is given by a population size of 100 000, as data is provided on incidence per 100 000 level. Of the population, 3280 are initially infected (first data point), 20 000 initially recovered, and the remaining share of the population being susceptible.

We first fit a Gaussian curve to each season's data series. From these fits we extracted the values of t_i and σ_i and treated them as fixed. The observation noise level is fixed to $\sigma = 0.4$. The other parameters are then estimated using the PMMH method using 50 particles and 10 000 iterations of the outer MH scheme. The parameters with bounds are given in Table 1.

D.1.2 ODE-SEIRDB model for Ebola

We use an SEIRDB model based on the SEIHDR model presented in Legrand et al. [24], explicitly using a dead but not-buried compartment for postmortem transmission. The formulation used in the manuscript is given by the following set of equations

$$\frac{\mathrm{d}S}{\mathrm{d}t} = -(\rho_I I + \rho_D D)S,$$

Table 1: **Parameters with bounds for SDE SIRS influenza model in Fig. 4b.**

Symbol	Description	Support	Initial value
β_0	Baseline transmission rate	[0.01, 0.5]	0.05
T_I	Days being infected	$[1.0, 14.0]$	7.0
T_R	Days being immune	$[1.0, 50.0]$	14.0
κ_1	Seasonal influence season 1	$[0.1, 0.5]$	0.2
κ_2	Seasonal influence season 2	$[0.1, 0.5]$	0.2
κ_3	Seasonal influence season 3	$[0.1, 0.5]$	0.2

$$\begin{aligned}
\frac{\mathrm{d}E}{\mathrm{d}t} &= (\rho_I I + \rho_D D)S - \frac{1}{T_E}E, \\
\frac{\mathrm{d}I}{\mathrm{d}t} &= \frac{1}{T_E}E - \frac{1}{T_I}I, \\
\frac{\mathrm{d}R}{\mathrm{d}t} &= \frac{\mu_I^R}{T_I}I, \\
\frac{\mathrm{d}D}{\mathrm{d}t} &= \frac{1-\mu_I^R}{T_I}I - \frac{1}{T_B}D, \\
\frac{\mathrm{d}B}{\mathrm{d}t} &= \frac{1}{T_B}D.
\end{aligned}$$

The model is calibrated to the Ebola outbreak in Guinea in 2014 [25, 26]. The start day is January 1st, 2014 and the last day is December 31st, 2014.

As observables, we use the flows from $E \to I$ together with a negative binomial noise model

$$Y \sim NB(d, p) \quad \text{with} \quad p = X/(X + d),$$

where d is the dispersion parameter and p the success probability.

As initial state, we use a population size of 1×10^7, which is roughly the population size of Guinea and with 10 initially infected. All other individuals are designated as susceptible.

We fix the dispersion to $d = 5.0$ and estimate all other parameters jointly using the PMMH method described above with 50 particles and 10 000 iterations of the outer MH scheme. The parameters with bounds are given in Table 2.

Table 2: **Parameters with bounds for ODE SEIRDB Ebola model in Fig. 4c.**

Symbol	Description	Support	Initial value
ρ_I	Transmission probability on contact with infected	$[0.0001, 0.5]$	0.005
T_E	Days being exposed	$[1.0, 30.0]$	5.0
T_I	Days being infected	$[1.0, 50.0]$	4.0
μ_I^R	Probability to recover	$[0.01, 0.99]$	0.5
ρ_D	Transmission probability on contact with deceased	$[0.0001, 0.5]$	0.025
T_B	Time until buried	$[1.0, 20.0]$	2.0

D.2 Calibration with BayesFlow for spatio-temporal SARS-CoV-2

For early SARS-CoV-2 in autumn 2020 where neither vaccination nor waning immunity played a substantial role for disease dynamics, we use a SECIR-type model as presented in Kühn et al. [27]. In addition to a standard SEIR model, we use a pre-symptomatic infectious state as well as a severe (hospitalization) and a critical (ICU) state. The local model is described by the equations

$$\begin{aligned}
\frac{\mathrm{d}S}{\mathrm{d}t} &= -\frac{S}{N-D}\,\phi\,\rho\left(\xi_C\,C + \xi_I\,I\right), \\
\frac{\mathrm{d}E}{\mathrm{d}t} &= \frac{S}{N-D}\,\phi\,\rho\left(\xi_C\,C + \xi_I\,I\right) - \frac{1}{T_E}\,E, \\
\frac{\mathrm{d}C}{\mathrm{d}t} &= \frac{1}{T_E}\,E - \frac{1}{T_C}\,C,
\end{aligned}$$

$$
\begin{aligned}
\frac{\mathrm{d}I}{\mathrm{d}t} &= \frac{\mu_C^I}{T_C} C - \frac{1}{T_I} I, \\
\frac{\mathrm{d}H}{\mathrm{d}t} &= \frac{\mu_I^H}{T_I} I - \frac{1}{T_H} H, \\
\frac{\mathrm{d}U}{\mathrm{d}t} &= \frac{\mu_H^U}{T_H} H - \frac{1}{T_U} U, \\
\frac{\mathrm{d}R}{\mathrm{d}t} &= \frac{1-\mu_C^I}{T_C} C + \frac{1-\mu_I^H}{T_I} I + \frac{1-\mu_H^U}{T_H} H + \frac{1-\mu_U^D}{T_U} U, \\
\frac{\mathrm{d}D}{\mathrm{d}t} &= \frac{\mu_U^D}{T_U} U.
\end{aligned}
\tag{1}
$$

We employ amortized Bayesian inference through BayesFlow [16] for calibration of the model. Here, a neural inference model is trained exclusively on simulated data and then applied to real observations without requiring additional model-specific optimization. Once trained, posterior inference for the observed data y_{obs} is obtained by a forward pass through the network, enabling fast inference and uncertainty quantification in downstream applications.

The BayesFlow pipeline is composed of two main neural components: (i) a summary network which encodes the simulator output into a latent representation, and (ii) an inference network which transforms the latent representation into a posterior approximation. These two networks can be trained jointly to provide optimal latent representation for inference [28].

In this work, we consider the following architectures:

- Summary network: either a *TimeSeriesNetwork*, which is tailored to sequential model outputs, or a *FusionTransformer*, which provides a flexible transformer-based encoder for structured time series.
- Inference network: a *FlowMatching* model, representing a state-of-the-art approach [15], capable of capturing complex posterior distributions in high-dimensional parameter spaces.

Table 3: **Prior distributions for the fitting of the ODE-based SECIR-type MPM in Fig. 4d,e.**

Symbol	Description	Distribution
	Factor correcting for underreported infections	Uniform(1., 10.)
$\bar{r}$	Mean contact reduction factor	Uniform(0., 1.)
r_i	State-specific contact reduction factor	TruncatedNormal(MEAN $= \bar{r}$, STD $= 0.1$)
T_E	Average time spent in Exposed state	Uniform(1., 5.2)
T_I	Average time spent in Infected state	Uniform(4., 10.)
T_H	Average time spent in Hospitalized state	Uniform(5., 10.)
T_U	Average time spent in ICU state	Uniform(9., 17.)
μ_C^R	Proportion of Recovered per Carrier	Uniform(0., 0.4)
μ_I^H	Proportion of Hospitalized per Infected	Uniform(0., 0.2)
μ_H^U	Proportion of ICU Cases per Hospitalized	Uniform(0., 0.4)
μ_U^D	Proportion of Dead per ICU	Uniform(0., 0.4)
ρ	Transmission probability on contact	Uniform(0., 0.2)

Table 4: **Overview of the network details for calibration of the models for Germany and Spain in Fig. 4d,e.**

**Scenario**	**Contact change time points**	**Summary Network**	**Inference Network**	Epochs
Germany (spatially resolved)	15, 30, 45	`FusionTransformer`	`FlowMatching`, width =512	500
Germany	15, 25, 35	`TimeSeriesNetwork`	`FlowMatching`, width =256	300
Spain (spatially resolved)	15, 30, 45	`TimeSeriesNetwork`	`FlowMatching`, width =512	500
Spain	0, 20, 40	`TimeSeriesNetwork`	`FlowMatching`, width =512	500

Training is performed using only simulated trajectories, without access to the observed dataset beyond its dimensionality and preprocessing conventions. After training, the resulting amortized posterior estimator q can be applied to y_{obs} to generate posterior samples $\theta \sim q(\theta \mid y_{\text{obs}})$. These posterior samples can then be simulated again to validate if estimated parameters fit the real data.

For the model calibration of Germany and Spain, we use the graph-based MPM with the deterministic mobility approach. The edge weights are derived from official registration and mobility data, respectively, for Germany [29] and for Spain [30]. We use reported data from October 1st, 2020 and before [31, 32, 33, 34] together with population data according to the respective spatial resolution to initialize the ODE-based models. The baseline contact rates are extracted from Prem et al. [35] and set to 7.95 for Germany and 12.32 for Spain. We assume symptomatic and asymptomatic individuals to be equally infectious, i.e., $\xi_C = \xi_I = 1$. The contact rates are adjusted every 10 to 20 days, representing the implementation of nonpharmaceutical interventions (NPIs) or behavioral adaptation. For the models with spatial resolution, NPIs and adaptation are always implemented on a federal state level. The strictness of the NPIs as well as the majority of epidemiological parameters are calibrated to the number of ICU cases [31, 33] by neural parameter estimation through BayesFlow [16]. For the model of Spain, we use version 2.0.7, while the model of Germany is calibrated using the updated version 2.0.8. We assume uniform prior distributions for the epidemiological parameters. Their bounds can be found in Table 3. For the damping values, we assume that the strength of the NPI is similar across the country. Therefore, we draw a mean value from a uniform distribution and then draw the strength for every federal state from a truncated normal distribution with this mean. Observation noise is modeled additively on top of the deterministic model output using a spike-and-slab distribution [36] with a spike of 0.4 and a slab of 0.2. Table 4 gives an overview over the networks and details for each of the scenarios. For the summary network, we set the dropout probability to 0.1, and the summary dimension to twice the number of parameters. For the inference network, we only change the width of the backbone of the flow matching model. All other hyperparameters are set to their defaults in BayesFlow.

E Models and parameters for scenario analysis of NPIs

In this section, we describe the scenario analysis of Fig. 4f-i. Building upon the calibrated graph-based MPM described in Section D.2, we study the effects of different NPI strategies in a scenario analysis. To select a representative simulation, we use the simulation with the smallest root mean square deviation. The corresponding parameters can be found in Table 5. Selecting the system state at day 60 (November 30th, 2020) as a starting point, we investigate the system's behavior under the following scenarios:

- No NPIs: lift all NPIs after day 60.
- NPIs continued: continue with the calibrated NPIs at day 60.
- Strict NPIs: increase calibrated NPI strictness by a factor of 1.2.
- DynamicNPIs: introduce DynamicNPIs at day 60 for incidence thresholds of 250 and 1 000 with a strictness of 0.63 and 0.77, respectively. DynamicNPIs are enforced for 14 days if a threshold is exceeded and continued if the simulated numbers stay above the threshold.
- DynamicNPIs with optimal control: formulate an optimal control problem with the strictness of DynamicNPIs as control parameters. The resulting strictness values of 0.53 und 0.9 are obtained by constraining total infections on January 21, 2021 to 500 000 and minimizing an objective function that penalizes NPI strictness if NPIs are active, i.e., when the corresponding incidence thresholds of 250 and 1 000 are exceeded, scaled by regional population shares and aggregated across regions and time. For more details on optimal control, see Section B.3.

F Models and parameters for model comparison

In this section we present the models used for the temporal-hybrid model comparison (Fig. 5b-c, Fig. 6d) and the ones used for the ABM, ODE, LCT and IDE comparison (Fig. 5d-e).

F.1 Temporal-hybrid model comparison and scaling

For the applications of the temporal-hybrid in Fig. 5b-c and Fig. 6d, the diffusive ABM (dABM) is coupled with the ODE-based SECIR-type model. The ODE-based SECIR-type model is given by Eq. (1). For the dABM, the same infection states and state transitions are used, however infectious agents can transmit the virus only to agents within a given interaction radius $r \in \mathbb{R}$. Individual movement is defined

Table 5: Parameters for NPI scenarios in Fig. 4f-i.

Symbol	Description	Value
T_E	Average time spent in Exposed state	4.88165
T_C	Average time spent in Carrier state	0.31835
T_I	Average time spent in Infected state	5.55430
T_H	Average time spent in Hospitalized state	9.32235
T_U	Average time spent in ICU state	15.49097
μ_C^R	Proportion of Recovered per Carrier	0.19291
μ_I^H	Proportion of Hospitalized per Infected	0.00450
μ_H^U	Proportion of ICU Cases per Hospitalized	0.17786
μ_U^D	Proportion of Dead per ICU	0.15412
ρ	Transmission probability on contact	0.09050

by diffusion processes on the single well potential

$$F(x,y) = \frac{x^4 + y^4}{2},$$

enforcing agents to stochastically return to a center region and interact with each other. The temporal-hybrid model switches between two models according to a predefined condition. With $m_1(t) \in \Omega_1$ the system state of the first model at time t and $m_2(t) \in \Omega_2$ the system state of the second model, respectively, the condition is defined by a function $\Gamma : \Omega_1 \times \Omega_2 \to \{0,1\}$. If $\Gamma\left(m_1(t), m_2(t)\right) = 1$, a model switch is triggered and the state of the currently active model has to be converted to the next used model using appropriate conversion functions. For the applications in this paper, a threshold value τ is used and Γ is given by

$$\Gamma\left(m_1(t), 0\right) = \begin{cases} 1, & \text{if } \left(I_{total}(t) > \tau \vee I_{total}(t) < 1\right) \\ 0, & \text{else} \end{cases}$$

with $m_1(t)$ the system state of the dABM at time t and $I_{total}(t) = E(t) + C(t) + I(t) + H(t) + U(t)$ the total number of infected agents at time t. For the application in Fig. 5b-c a threshold of three infected agents ($\tau = 3$) is used and for Fig. 6d a threshold of 2 %, 5 % and 10 % ($\tau \in \{0.02n_a, 0.05n_a, 0.1n_a\}$) is compared where n_a denotes the number of agents.
Since the ODE-SECIR model lacks spatial resolution, the dABM state can be converted by aggregating agents to subpopulations according to their infection state. Conversely, converting the ODE-SECIR state to the dABM would require reconstruction of agents' positions and infection states which could be done via random sampling. However, in the presented scenarios, the temporal-hybrid model does not switch back once its active model is the ODE-based model. All parameters used for the simulations are given in Table 6.

F.2 Comparison of ABM and ODE-, LCT- and IDE-based models

For the comparison of the mobility-based ABM and the ODE-, LCT- and IDE-based models, we consider a SECIR-type disease progression. For more realistic assumptions, we use age-resolved models. To simplify notation, we will describe the used models only for one age group in the following; see Table 7 and Table 8 for a description of all used parameters.

The ODE-based SECIR-type model is given by Eq. (1). The LCT-based SECIR-type model is given by

$$\begin{aligned}
\frac{\mathrm{d}S}{\mathrm{d}t} &= -\frac{S}{N-D}\,\phi\,\rho\left(\xi_C\,C_* + \xi_I\,I_*\right), \\
\frac{\mathrm{d}E_1}{\mathrm{d}t} &= \frac{S}{N-D}\,\phi\,\rho\left(\xi_C\,C_* + \xi_I\,I_*\right) - \frac{n_E}{T_E}\,E_1, \\
\frac{\mathrm{d}E_j}{\mathrm{d}t} &= \frac{n_E}{T_E}\,E_{j-1} - \frac{n_E}{T_E}\,E_j, && \text{for } j \in \{2,\dots,n_E\} \\
\frac{\mathrm{d}C_1}{\mathrm{d}t} &= \frac{n_E}{T_E}\,E_{n_E} - \frac{n_C}{T_C}\,C_1,
\end{aligned}$$

Table 6: **Parameters for temporal-hybrid applications in Fig. 5b-c and Fig. 6d.**

Symbol	Description	Value
n_a	Number of agents	10 000 (Fig. 5b-c), $\{100, 1\,000, 5\,000, 10\,000, 20\,000, 30\,000\}$ (Fig. 6d)
μ_C^I	Proportion of Infected per Carrier	0.75
μ_I^H	Proportion of Hospitalized per Infected	0.01
μ_H^U	Proportion of ICU Cases per Hospitalized	0.1
μ_U^D	Proportion of Dead per ICU	0.05
T_E	Average time spent in Exposed state	4.5
T_C	Average time spent in Carrier state	3
T_I	Average time spent in Infected state	7
T_H	Average time spent in Hospitalized state	15
T_U	Average time spent in ICU state	16
λ	Transmission rate	0.5
r	dABM interaction radius	0.1
σ	dABM noise parameter	0.5
$E(t_0)$	Initially Exposed	1 (Fig. 5b-c), 1 % (Fig. 6d)
t_0	Simulation start	0
dt	(Initial) step size	0.1
$t_{\max}$	Simulation time frame in days	90 (Fig. 5b-c), 60 (Fig. 6d)

$$\begin{aligned}
\frac{\mathrm{d}C_j}{\mathrm{d}t} &= \frac{n_C}{T_C}\,C_{j-1} - \frac{n_C}{T_C}\,C_j, && \text{for } j \in \{2,\dots,n_C\}\\
\frac{\mathrm{d}I_1}{\mathrm{d}t} &= \mu_C^I\,\frac{n_C}{T_C}\,C_{n_C} - \frac{n_I}{T_I}\,I_1,\\
\frac{\mathrm{d}I_j}{\mathrm{d}t} &= \frac{n_I}{T_I}\,I_{j-1} - \frac{n_I}{T_I}\,I_j, && \text{for } j \in \{2,\dots,n_I\}\\
\frac{\mathrm{d}H_1}{\mathrm{d}t} &= \mu_I^H\,\frac{n_I}{T_I}\,I_{n_I} - \frac{n_H}{T_H}\,H_1,\\
\frac{\mathrm{d}H_j}{\mathrm{d}t} &= \frac{n_H}{T_H}\,H_{j-1} - \frac{n_H}{T_H}\,H_j, && \text{for } j \in \{2,\dots,n_H\}\\
\frac{\mathrm{d}U_1}{\mathrm{d}t} &= \mu_H^U\,\frac{n_H}{T_H}\,H_{n_H} - \frac{n_U}{T_U}\,U_1,\\
\frac{\mathrm{d}U_j}{\mathrm{d}t} &= \frac{n_U}{T_U}\,U_{j-1} - \frac{n_U}{T_U}\,U_j, && \text{for } j \in \{2,\dots,n_U\}\\
\frac{\mathrm{d}R}{\mathrm{d}t} &= \left(1-\mu_C^I\right)\frac{n_C}{T_C}\,C_{n_C} + \left(1-\mu_I^H\right)\frac{n_I}{T_I}\,I_{n_I},\\
&\quad + \left(1-\mu_H^U\right)\frac{n_H}{T_H}\,H_{n_H} + \left(1-\mu_U^D\right)\frac{n_U}{T_U}\,U_{n_U},\\
\frac{\mathrm{d}D}{\mathrm{d}t} &= \mu_U^D\,\frac{n_U}{T_U}\,U_{n_U},
\end{aligned} \tag{2}$$

where $Z_* = \sum_{j=1}^{n_Z} Z_j$ is the total number of individuals in compartment Z.

The flow-based formulation of the IDE-based SECIR-type model is given by

$$\begin{aligned}
\frac{\mathrm{d}S}{\mathrm{d}t} &= -\sigma_{S,E},\\
\frac{\mathrm{d}E}{\mathrm{d}t} &= \sigma_{S,E} - \sigma_{E,C},\\
\frac{\mathrm{d}C}{\mathrm{d}t} &= \sigma_{E,C} - \sigma_{C,I} - \sigma_{C,R},\\
\frac{\mathrm{d}I}{\mathrm{d}t} &= \sigma_{C,I} - \sigma_{I,H} - \sigma_{I,R},\\
\frac{\mathrm{d}H}{\mathrm{d}t} &= \sigma_{I,H} - \sigma_{H,U} - \sigma_{H,R},
\end{aligned} \tag{3}$$

$$
\begin{aligned}
\frac{\mathrm{d}U}{\mathrm{d}t} &= \sigma_{H,U} - \sigma_{U,D} - \sigma_{U,R},\\
\frac{\mathrm{d}R}{\mathrm{d}t} &= \sigma_{C,R} + \sigma_{I,R} + \sigma_{H,R} + \sigma_{U,R},\\
\frac{\mathrm{d}D}{\mathrm{d}t} &= \sigma_{U,D},
\end{aligned}
$$

where the flows are defined via

$$
\begin{aligned}
\sigma_{S,E}(t) &= S(t)\,\lambda(t), & \sigma_{E,C}(t) &= -\int_{-\infty}^{t} {\gamma_E^C}'(t-x)\,\sigma_{S,E}(x)\,\mathrm{d}x,\\
\sigma_{C,I}(t) &= -\,\mu_C^I \int_{-\infty}^{t} {\gamma_C^I}'(t-x)\,\sigma_{E,C}(x)\,\mathrm{d}x, & \sigma_{C,R}(t) &= -\left(1-\mu_C^I\right)\int_{-\infty}^{t} {\gamma_C^R}'(t-x)\,\sigma_{E,C}(x)\,\mathrm{d}x,\\
\sigma_{I,H}(t) &= -\,\mu_I^H \int_{-\infty}^{t} {\gamma_I^H}'(t-x)\,\sigma_{C,I}(x)\,\mathrm{d}x, & \sigma_{I,R}(t) &= -\left(1-\mu_I^H\right)\int_{-\infty}^{t} {\gamma_I^R}'(t-x)\,\sigma_{C,I}(x)\,\mathrm{d}x,\\
\sigma_{H,U}(t) &= -\,\mu_H^U \int_{-\infty}^{t} {\gamma_H^U}'(t-x)\,\sigma_{I,H}(x)\,\mathrm{d}x, & \sigma_{H,R}(t) &= -\left(1-\mu_H^U\right)\int_{-\infty}^{t} {\gamma_H^R}'(t-x)\,\sigma_{I,H}(x)\,\mathrm{d}x,\\
\sigma_{U,D}(t) &= -\,\mu_U^D \int_{-\infty}^{t} {\gamma_U^D}'(t-x)\,\sigma_{H,U}(x)\,\mathrm{d}x, & \sigma_{U,R}(t) &= -\left(1-\mu_U^D\right)\int_{-\infty}^{t} {\gamma_U^R}'(t-x)\,\sigma_{H,U}(x)\,\mathrm{d}x,
\end{aligned}
$$

and the force of infection is given by

$$
\begin{aligned}
\lambda(t) = \frac{\phi(t)}{N-D(t)} \int_{-\infty}^{t} & \xi_C\,\rho\left(\mu_C^I\,\gamma_C^I(t-x) + \left(1-\mu_C^I\right)\gamma_C^R(t-x)\right)\sigma_{E,C}(x)\\
&+ \xi_I\,\rho\left(\mu_I^H\,\gamma_I^H(t-x) + \left(1-\mu_I^H\right)\gamma_I^R(t-x)\right)\sigma_{C,I}(x)\;\mathrm{d}x.
\end{aligned}
$$

The function $\gamma_{Z_1}^{Z_2}$ denotes the survival function of the distribution with respect to $T_{Z_1}^{Z_2}$, i.e. the mean time spent in state Z_1 before transitioning to state Z_2.

In setup S1, exponential infection state transition distributions are used for all models, and we additionally assume $T_{Z_1}^{Z_2} = T_{Z_1}$ for all feasible Z_2 for the ABM and IDE-based model. In setup S2, lognormal state transition distributions are used for the ABM and IDE-based model based on representative data for COVID-19 [37]. For the LCT-based model, the parameters of the Erlang distribution are chosen such that mean and variance are as close as possible to the corresponding lognormal distribution. The exponential distributions in the ODE-based model are chosen such that the mean values agree with those of the corresponding lognormal distributions. For both setups, we additionally compared different location structures for the ABM. While in Setups S1.1 and S2.1 only one location per type is used, corresponding to homogeneous mixing in every location type, in Setups S1.2 and S2.2 multiple locations per type are used, see Table 8 for the applied distributions.

To ensure comparability between different models, we simulated a single trajectory with the ABM for 20 days to initialize all models with the same state. More precisely, we use the sample infection dynamics to initialize the ABM and IDE-based model whereas for the LCT- and the ODE-based model only the compartment sizes on day 20 are used. The transmission probability on contact used in the ODE-, LCT- and IDE-based models is harmonized with the transmission rates of the ABM based on S1.1 and subsequently used for all other scenarios. Eventually, simulations of all models are run for 100 days. The comparison is done between 100 ABM and one deterministic run for each PBM, all starting from day 20. The effective reproduction number as provided on day 20 is computed with the ODE-based model using the next generation matrix.

Table 7: **Joint parameters for all scenarios for ABM-IDE-LCT-ODE comparison application in Fig. 5d-e.**

Symbol	Scenario	Description	Value
N_G	all	Number of age groups	6
W_{age}	all	Age group distribution	$(0.0477, 0.0903, 0.2275, 0.3447, 0.2183, 0.0714)$
μ_C^I	all	Proportion of Infected per Carrier	$(0.75, 0.75, 0.8, 0.8, 0.8, 0.8)$
μ_I^H	all	Proportion of Hospitalized per Infected	$(0.0075, 0.0075, 0.019, 0.0615, 0.165, 0.225)$
μ_H^U	all	Proportion of ICU cases per Hospitalized	$(0.075, 0.075, 0.075, 0.15, 0.3, 0.4)$
μ_U^D	all	Proportion of Dead per ICU	$(0.05, 0.05, 0.14, 0.14, 0.4, 0.6)$
ϕ	all	Contact matrix between age groups	Based on Prem et al. [35] and Fumanelli et al. [38]
ρ	all	Transmission probability on contact	(0.0409163, 0.0351982, 0.0337711, 0.0333412, 0.0328736, 0.0300238)
ξ_C	all	Proportion of Carrier individuals not isolated	1
ξ_I	all	Proportion of Infected individuals not isolated	1
v_P	all	Viral load peak for ABM	8.1
v_I	all	Viral load incline for ABM	2
v_D	all	Viral load decline for ABM	-0.17
(α, β)	all	Viral shed parameters for ABM	$(-7, 1)$
s_f	all	Viral shed factor for ABM	Uniform$(0, 0.6)$

Table 8: **Scenario-specific parameters for ABM-IDE-LCT-ODE comparison application in Fig. 5d-e.**

**Symbol**	**Scenario**	**Description**	**Value**
T_E	S1	Average time spent in Exposed state	$(4.5, 4.5, 4.5, 4.5, 4.5, 4.5)$
T_C	S1	Average time spent in Carrier state	$(2.825, 2.825, 2.48, 2.48, 2.48, 2.48)$
T_I	S1	Average time spent in Infected state	$(7.9895, 7.9895, 7.9734, 7.9139, 7.9139, 7.685)$
T_H	S1	Average time spent in Hospitalized state	$(16.855, 16.855, 16.855, 15.61, 13.12, 11.46)$
T_U	S1	Average time spent in ICU state	$(17.73, 17.73, 17.064, 17.064, 15.14, 13.66)$
T_E^C	S2	Time from E to C	$\text{LogNormal}(\text{MEAN} = 4.5, \text{STD} = 1.5)$
T_C^I	S2	Time from C to I	$\text{LogNormal}(\text{MEAN} = 1.1, \text{STD} = 0.9)$
T_C^R	S2	Time from C to R	$\text{LogNormal}(\text{MEAN} = 8.0, \text{STD} = 2.0)$
T_I^H	S2	Time from I to H	$\text{LogNormal}(\text{MEAN} = 6.6, \text{STD} = 4.9)$
T_I^R	S2	Time from I to R	$\text{LogNormal}(\text{MEAN} = 8.0, \text{STD} = 2.0)$
T_H^U	S2	Time from H to U	$\text{LogNormal}(\text{MEAN} = 1.5, \text{STD} = 2.0)$
T_H^R	S2	Time from H to R	$\text{LogNormal}(\text{MEAN} = 18.1, \text{STD} = 6.3)$
T_U^D	S2	Time from U to D	$\text{LogNormal}(\text{MEAN} = 10.7, \text{STD} = 4.8)$
T_U^R	S2	Time from U to R	$\text{LogNormal}(\text{MEAN} = 18.1, \text{STD} = 6.3)$
n_E	S2	Number of subcompartments in the LCT model for E	$(9, 9, 9, 9, 9, 9)$
n_C	S2	Number of subcompartments in the LCT model for C	$(5, 5, 4, 4, 4, 4)$
n_I	S2	Number of subcompartments in the LCT model for I	$(16, 16, 16, 15, 15, 13)$
n_H	S2	Number of subcompartments in the LCT model for H	$(8, 8, 8, 7, 6, 5)$
n_U	S2	Number of subcompartments in the LCT model for U	$(8, 8, 8, 8, 7, 6)$
$W_{household}$	S1.2, S2.2	Household size distribution for the ABM	$(0.415, 0.342, 0.118, 0.091, 0.034)$
$n_{workplace}$	S1.2, S2.2	Workplace size for the ABM	$\text{Normal}(\text{MEAN} = 15, \text{STD} = 25)$
$n_{workplace}^{min}$	S1.2, S2.2	Minimum workplace size for the ABM	5
n_{school}	S1.2, S2.2	School size for the ABM	$\text{Normal}(\text{MEAN} = 42, \text{STD} = 3)$
n_{school}^{min}	S1.2, S2.2	Minimum school size for the ABM	15
n_{Event}	S1.2, S2.2	Event size for the ABM	$\text{Normal}(\text{MEAN} = 42, \text{STD} = 941)$
n_{Event}^{min}	S1.2, S2.2	Minimum event size for the ABM	10
n_{Shop}	S1.2, S2.2	Shop size for the ABM	$\text{Normal}(\text{MEAN} = 90, \text{STD} = 1000)$
n_{Shop}^{min}	S1.2, S2.2	Minimum shop size for the ABM	30

G Cross-implementation performance: C++, Python, and R

This section details the implementation specifics for the runtime comparison presented in Fig. 6a of the main manuscript. The objective of this benchmark is to quantify the computational efficiency of MEmilio's C++ backend – accessed directly and via its Python interface – against standard implementation patterns in the statistical computing language R; which is often used for epidemiological modeling (see Fig. 1b of the manuscript).

G.1 Model specification and metapopulation extension

For this benchmark, we utilize the standard SEIR compartmental model without age stratification. The local dynamics for a population of size $N(t) = S(t) + E(t) + I(t) + R(t)$ are given by the following system of ODEs with

$$\begin{aligned}\frac{\mathrm{d}S}{\mathrm{d}t} &= -\lambda S,\\ \frac{\mathrm{d}E}{\mathrm{d}t} &= \lambda S - \frac{1}{T_E} E,\\ \frac{\mathrm{d}I}{\mathrm{d}t} &= \frac{1}{T_E} E - \frac{1}{T_I} I,\\ \frac{\mathrm{d}R}{\mathrm{d}t} &= \frac{1}{T_I} I.\end{aligned}$$

Here, $T_E \in \mathbb{R}_{>0}$ and $T_I \in \mathbb{R}_{>0}$ denote the average latency and infectious periods, respectively. The force of infection λ_i is defined as

$$\lambda = \rho \phi \frac{I}{N},$$

where $\rho \in [0, 1]$ is the transmission probability and $\phi \in \mathbb{R}_{>0}$ denotes the mean daily contact rate.

To evaluate scalability, we extend the PBM to an MPM using the ODE-integrated mobility scheme as presented in the methods section. The resulting global system of ODEs has a dimension of $4N_P$, where N_P is the number of patches. The benchmark scales the number of modeled regions N_P from 1 to 256. To ensure a computational load on the mobility handling, we assumed a fully connected topology where individuals can infect from infected individuals of all regions; weighted with the corresponding commuter exchange. The mobility coefficients are initialized uniformly.

G.2 Implementations

We compare four implementation approaches to solve the resulting system of ODEs:

1. **MEmilio C++:** Direct execution of the compiled C++ library using both the explicit Euler and the Runge-Kutta-4 (RK4) integrator.
2. **MEmilio Python (with precompiled C++ code):** Execution via MEmilio's Python bindings. This setup uses the identical C++ core for calculation but handles the initialization and function calls through Python, testing the overhead introduced by the Python interface layer.
3. **R (Pure):** A native R implementation of the explicit Euler scheme. This represents a baseline for ad-hoc scripts as used for rapid prototyping or modeling tasks.
4. **R (with precompiled deSolve):** An implementation using the deSolve package in R, which uses compiled C/Fortran code for the integration steps.

G.3 Simulation parameters

All simulations are performed over a time horizon of $T = 500$ days with a fixed step size of $\Delta t = 0.1$. The initial parameters, epidemiological rates, and population sizes used for this benchmark are identical across all implementations to ensure comparability and the values are listed in Table 9. To mitigate the impact of execution variability, each benchmark configuration is repeated 40 times. We report the median runtime to ensure stable timings robust against outliers. The simulations are executed on a dedicated compute node equipped with an Intel Xeon Skylake CPU (Gold 6132) with 2.60 GHz and 384 GB DDR4 memory.

Table 9: Simulation parameters and initial conditions for the cross-implementation performance benchmark in Fig. 6a. The settings are applied identically across the C++, Python, and R implementations to ensure the comparability of the runtime results.

Symbol	Description	Value
N_G	Number of age groups (non-stratified)	1
N_P	Number of patches (regions) scaled for the benchmark	$\{1, 2, \ldots, 256\}$
$S^{(r)}(t_0)$	Initial susceptible population per region r	10 000
$E^{(0)}(t_0)$	Initial exposed individuals in region 0 (patient zero seed)	100
$E^{(r)}(t_0)$	Initial exposed individuals in regions $r > 0$	0
$I^{(r)}(t_0)$	Initial infected individuals (all regions)	0
$R^{(r)}(t_0)$	Initial recovered individuals (all regions)	0
ϕ	Average number of daily contacts	2.7
ρ	Transmission probability on contact	0.01
T_E	Average time spent in Exposed state	5.2 days
T_I	Average time spent in Infected state	6.0 days
$t_{\max}$	Simulation time frame in days	500
Δt	Fixed step size for numerical integration	0.1 days
C_r	Retention rate (individuals staying in home patch r)	0.8
C_{rs}	Mobility rate ($r \neq s$): Outflow distributed uniformly to all other patches (fully connected)	$\frac{0.2}{N_P-1}$

H Benchmarking Graph Neural Network surrogates against metapopulation model

This section describes the setup for the performance comparison between the mechanistic graph-based MPM based on the formulation in Kühn et al. [27] and the Graph Neural Network (GNN) surrogate model based on Schmidt et al. [39], as presented in Fig. 6e of the main manuscript. The objective is to quantify the computational speedup achieved by replacing the numerical integrator with a learned surrogate in large-scale ensemble scenarios.

H.1 Surrogate model architecture and training

The surrogate model is implemented using TensorFlow [40] and the Spektral [41] library for graph deep learning. The architecture is adapted from Schmidt et al. [39] and designed to capture spatio-temporal dynamics on the graph of Germany's 400 districts ($N = 400$) considering $N_G = 6$ distinct age groups.

Specifically, the network consists of a GNN with seven ARMA convolutional layers (ARMAConv) [42], each configured with 512 channels and ReLU activation. The final layer is a dense projection layer that maps the node features to the output time-series (flattened trajectory of all compartments over the simulation horizon). Input features include the binary connectivity matrix of the mobility network and the full initial state vector, comprising eight epidemiological compartments for each of the six age groups across all 400 districts.

To ensure the surrogate generalizes across different epidemiological regimes, it is trained on a dataset of 1 000 distinct mechanistic simulations. These simulations are generated by varying the initial conditions, specifically the magnitude and spatial distribution of the initial infections, in a graph-based MPM. The dataset is split into training (80 %), validation (10 %), and test (10 %) sets. Training is performed using the Adam optimizer with a mean squared error loss function. To ensure robust convergence and prevent overfitting, we employ early stopping based on the validation loss. Detailed information on the parameter distributions used for dataset generation can be found in Schmidt et al [39].

H.2 Benchmark setup

We compare the runtime of both approaches for three distinct prediction horizons (30, 60, and 90 days) and varying simulation batch sizes $B \in \{1, 2, 4, \ldots, 128\}$. The simulation batch size represents the number of independent simulations computed simultaneously.

- **Graph-based MPM:** The reference runtimes are derived from the execution time of the optimized C++ solver for the graph-based MPM using a SECIR model (see Eq. Eq. (1)) for the local nodes. Since the numerical integration of independent ensemble members is performed sequentially on the benchmark hardware, the total runtime scales linearly with the number of simulations.

- **GNN Surrogate:** The evaluation time is measured on a GPU-accelerated node. For each configuration, we performed 100 evaluations and reported the mean wall-clock time. Thanks to the parallelism of the GPU, the evaluation time is nearly constant for all batch sizes.

The surrogate maintains a stable mean absolute percentage error of up to 15 % relative to the mechanistic ground truth across all batch sizes, confirming that the massive speedup does not come at the cost of degrading approximation quality [39]. All measurements are conducted on a compute node equipped with an Intel Xeon Scalable Processor Skylake (Silver) and four NVIDIA Tesla V100 SXM2 GPUs (16 GB VRAM). The GNN inference utilizes a single GPU, while the ODE baselines are executed on the CPU.

I Performance evaluation of PBMs and MPMs

In this section, we provide the models and setups used for the performance evaluation of the PBMs as presented in Fig. 6b,f of the manuscript.

I.1 Scaling with respect to regions in ODE-, LCT-, and IDE-based MPM

For the region-dependent scaling in Fig. 6b we use the graph-based SECIR-type MPM using ODE-based (see Eq. (1)), LCT-based (see Eq. (2)), and IDE-based (see Eq. (3)) models in the local nodes. For each model we consider $N_G = 1$ age groups and performed 100 simulations for a time frame of 30 days and a varying number of regions $N_P = \{200, 250, 300, ..., 1\,000\}$. The ODE- and LCT-based MPM realize mobility using the instant mobility approach with a node degree of 100. MEmilio's IDE-based MPM is not yet able to incorporate mobility, hence its simulations are run without any exchange of individuals between regions.

I.2 Strong scaling with respect to the number of simulations

The strong scaling setup for the PBMs in Fig. 6f uses SECIR-type models considering $N_G = 6$ age groups. The models are initialized with population and case data for the early phase of the COVID-19 pandemic in Germany [32] and with parameters motivated by Kühn et al. [27], with an added variation of ±10 % to all infection parameters such that the simulated scenarios differ in each run. The graph-based MPMs using ODEs represents $N_P = 400$ German counties while the simulations of IDE- and LCT-based model only use one global representation i.e., $N_P = 1$. Contact patterns for the graph-based MPM and LCT-based models are based on Prem et al. and Fumanelli et al. [35, 38]. The mobility data for the ODE-based MPM is obtained from official data from 2020 [43]. The parameter values for all models are detailed in Table 10. We performed a total of 16 384 simulations for the LCT- and IDE-based model each and 1 280 simulations for the graph-based MPM, scaled over 1 to 128 cores.

All PBM and MPM simulations are performed on (up to) three Intel Xeon Skylake (Gold 6132) CPU nodes with 56 cores with 2.60 GHz and 384 GB DDR4 memory.

Table 10: Parameters for scaling applications of PBMs and MPMs in Fig. 6b,f. Values in parentheses indicate values per age group (youngest to oldest).

**Symbol**	**Model**	**Description**	**Value**
N_G	all	Number of age groups	1 (Fig. 6b), 6 (Fig. 6f)
ξ_C	ODE, LCT	Proportion of people from the Carrier compartment who are not isolated	1.0
ξ_I	ODE, LCT	Proportion of people from the Infected compartment who are not isolated	0.3
ρ	ODE, LCT	Transmission probability on contact	0.07333 (Fig. 6b), $(0.03, 0.06, 0.06, 0.06, 0.09, 0.175)$ (Fig. 6f)
μ_C^R	ODE, LCT	Proportion of Recovered per Carrier	0.206901 (Fig. 6b), $(0.25, 0.25, 0.2, 0.2, 0.2, 0.2)$ (Fig. 6f)
μ_I^H	ODE, LCT	Proportion of Hospitalized per Infected	0.07864 (Fig. 6b), $(0.0075, 0.0075, 0.019, 0.0615, 0.165, 0.225)$ (Fig. 6f)
μ_H^U	ODE, LCT	Proportion of ICU cases per Hospitalized	0.17318 (Fig. 6b), $(0.075, 0.075, 0.075, 0.15, 0.3, 0.4)$ (Fig. 6f)
μ_U^D	ODE, LCT	Proportion of Dead per ICU	0.21718 (Fig. 6b), $(0.05, 0.05, 0.14, 0.14, 0.4, 0.6)$ (Fig. 6f)
T_E	ODE, LCT	Average time spent in Exposed state	3.335 (Fig. 6b), $(3.335, 3.335, 3.335, 3.335, 3.335, 3.335)$ (Fig. 6f)
T_C	ODE, LCT	Average time spent in Carrier state	2.58916 (Fig. 6b), $(2.74, 2.74, 2.565, 2.565, 2.565, 2.565)$ (Fig. 6f)
T_I	ODE, LCT	Average time spent in Infected state	6.94547 (Fig. 6b), $(7.02625, 7.02625, 7.0665, 6.9385, 6.835, 6.775)$ (Fig. 6f)
T_H	ODE, LCT	Average time spent in Hospitalized state	7.28196 (Fig. 6b), $(5., 5., 5.925, 7.55, 8.5, 11.)$ (Fig. 6f)
T_U	ODE, LCT	Average time spent in ICU state	13.066 (Fig. 6b), $(6.95, 6.95, 6.86, 17.36, 17.1, 11.6)$ (Fig. 6f)
n_Z	LCT	Number of subcompartments per compartment	1 (S, R, D), 5 (E, C, I, H, U)
$\gamma(\tau)$	IDE	Expected proportion of individuals who remain in the considered compartment τ days after entering	SmootherCosine(2)
ρ	IDE	Transmission probability on contact	ConstantFunction(1)
ξ_C	IDE	Proportion of people from the Carrier compartment who are not isolated	ConstantFunction(1)
ξ_I	IDE	Proportion of people from the Infected compartment who are not isolated	ConstantFunction(1)

Table 11: ABM Parameters for Fig. 6c,f. For the proportional parameters in the second part, values are given for the different age groups in ascending order. We denote LogNormal distributions with dependency on (mean, std) as parameters. Contact scaling of the baseline contact matrices are provided for the five location types: Home, School, Work, SocialEvent, and BasicsShop.

Symbol	Description	Value	Source
v_{P}	Viral load peak	8.1	[44]
v_{I}	Viral load incline	2	[44]
v_{D}	Viral load decline	-0.17	[44]
(α, β)	Viral shed parameters	$(-7, 1)$	[44]
s_{f}	Viral shed factor	Uniform$(0.00, 0.28)$	Adopted from [45]
s	Contact Matrix Scaling	$(21.0, 4.8, 1.5, 14.4, 3.16)$	[46]
λ	Infection rate from viral shed	1.0	Manual Adoption
μ_C^I	Proportion of Infected per Carrier	$(0.5, 0.55, 0.6, 0.7, 0.83, 0.9)$	[37, Tab. 2]
μ_I^H	Proportion of Hospitalized per Infected	$(0.02, 0.03, 0.04, 0.07, 0.17, 0.24)$	[47]
μ_H^U	Proportion of ICU cases per Hospitalized	$(0.1, 0.11, 0.12, 0.14, 0.33, 0.62)$	[48, Tab. 2]
μ_U^D	Proportion of Dead per ICU	$(0.12, 0.13, 0.15, 0.26, 0.4, 0.48)$	[48, Tab. 2]
T_E^C	Time from E to C	LogNormal$(\mathrm{MEAN} = 4.5, \mathrm{STD} = 1.5)$	[37, Tab. 1]
T_C^I	Time from C to I	LogNormal$(\mathrm{MEAN} = 1.1, \mathrm{STD} = 0.9)$	[37, Tab. 1]
T_C^R	Time from C to R	LogNormal$(\mathrm{MEAN} = 8.0, \mathrm{STD} = 2.0)$	[37, Tab. 1]
T_I^H	Time from I to H	LogNormal$(\mathrm{MEAN} = 6.6, \mathrm{STD} = 4.9)$	[37, Tab. 1]
T_I^R	Time from I to R	LogNormal$(\mathrm{MEAN} = 8.0, \mathrm{STD} = 2.0)$	[37, Tab. 1]
T_H^U	Time from H to U	LogNormal$(\mathrm{MEAN} = 1.5, \mathrm{STD} = 2.0)$	[37, Tab. 1]
T_H^R	Time from H to R	LogNormal$(\mathrm{MEAN} = 18.1, \mathrm{STD} = 6.3)$	[37, Tab. 1]
T_U^D	Time from U to D	LogNormal$(\mathrm{MEAN} = 10.7, \mathrm{STD} = 4.8)$	[37, Tab. 1]
T_U^R	Time from U to R	LogNormal$(\mathrm{MEAN} = 18.1, \mathrm{STD} = 6.3)$	[37, Tab. 1]

J Performance evaluation of the mobility-based ABM

The large-scale ABM simulations are executed on JURECA-DC supercomputing facility [49] equipped with compute nodes of 2x AMD EPYC 7742, 2× 64 cores with 512 GB DDR4 memory per node and where we fix the frequency to 2 GHz.

J.1 Comparisons with other frameworks

Runtime scaling experiments in Fig. 6c are conducted using the ABM configured with COVID-19 disease parameters as described in Table 11. The simulation is initialized with approximately 0.05 % of agents initially infected (exposed), using a synthetic population based on German census data [50, 51, 52, 53], divided into six age groups. To isolate computational overhead from logging operations, a minimal logger configuration is employed. Each benchmark run simulated five days of epidemic dynamics resulting in 120 time steps.

For comparison, equivalent runtime scaling experiments are performed using Covasim and OpenCOVID. Similarly to our ABM, simulations are initialized with approximately 0.05 % of the population initially infected (exposed) and run for 120 time steps of one day. Covasim benchmarks use the hybrid population generation for Germany and OpenCOVID benchmarks the default age distribution together with the default contact network for Germany. Finally, each simulation is run for 1, 2, 4, 8, 16, 32, 64, 128, and 256 million agents. Unfortunately, we were unable to complete simulations of Covasim and OpenCOVID with 128 and 256 million agents without running out of memory.

J.2 Strong scaling with respect to the number of simulations

Strong scaling in Fig. 6f is conducted using a population of 2 000 000 agents divided into six age groups. The synthetic population is generated based on German census data [50, 51, 52, 53], and the simulation is parameterized with COVID-19 disease parameters, again shown in 11. Results of 1 to 128 cores are conducted on a single node while results from 128 to 16 384 cores are conducted on 1 to 128 nodes with 128 cores each. As before, 0.05 % of agents are initially Exposed. Disease state transitions are recorded using a logger to capture the temporal evolution of epidemiological compartments. Each simulation covers a 14-day period.

References

[1] Ahnert, K. & Mulansky, M. Odeint – Solving Ordinary Differential Equations in C++. *AIP Conference Proceedings* **1389**, 1586–1589 (2011). URL https://doi.org/10.1063/1.3637934.

[2] Cash, J. R. & Karp, A. H. A variable order Runge-Kutta method for initial value problems with rapidly varying right-hand sides. *ACM Transactions on Mathematical Software* **16**, 201–222 (1990). URL https://doi.org/10.1145/79505.79507.

[3] Messina, E., Pezzella, M. & Vecchio, A. A non-standard numerical scheme for an age-of-infection epidemic model. *Journal of Computational Dynamics* **9**, 239 (2022). URL https://doi.org/10.3934/jcd.2021029.

[4] Wendler, A., Plötzke, L., Tritzschak, H. & Kühn, M. J. A nonstandard numerical scheme for a novel secir integro-differential equation-based model allowing nonexponentially distributed stay times. *Applied Mathematics and Computation* **509**, 129636 (2026). URL https://doi.org/10.1016/j.amc.2025.129636.

[5] Messina, E., Pezzella, M. & Vecchio, A. Positive Numerical Approximation of Integro-Differential Epidemic Model. *Axioms* **11**, 69 (2022). URL https://www.mdpi.com/2075-1680/11/2/69.

[6] Wendler, A., Hasenauer, J. & Kühn, M. Applied numerical analysis of higher-order schemes for integro-differential equation-based age-of-infection models with time-dependent contact rates (2026). To be submitted.

[7] Message Passing Interface Forum. *MPI: A Message-Passing Interface Standard Version 5.0* (2025). URL https://www.mpi-forum.org/docs/mpi-5.0/mpi50-report.pdf.

[8] Dagum, L. & Menon, R. OpenMP: an industry standard API for shared-memory programming. *Computational Science & Engineering, IEEE* **5**, 46–55 (1998). URL https://doi.org/10.1109/99.660313.

[9] Betts, J. T. *Practical methods for optimal control and estimation using nonlinear programming* (SIAM, 2010).

[10] Lin, F., Muthuraman, K. & Lawley, M. An optimal control theory approach to non-pharmaceutical interventions. *BMC Infectious Diseases* **10**, 32 (2010). URL https://doi.org/10.1186/1471-2334-10-32.

[11] Tsay, C., Lejarza, F., Stadtherr, M. A. & Baldea, M. Modeling, state estimation, and optimal control for the us covid-19 outbreak. *Scientific Reports* **10**, 10711 (2020). URL https://doi.org/10.1038/s41598-020-67459-8.

[12] Sass, S. & Mitsos, A. Obscured by terminology: Hidden parallels in direct methods for open-loop optimal control. *Journal of Process Control* **154**, 103513 (2025).

[13] Wächter, A. & Biegler, L. T. On the implementation of an interior-point filter line-search algorithm for large-scale nonlinear programming. *Mathematical programming* **106**, 25–57 (2006). URL https://doi.org/10.1007/s10107-004-0559-y.

[14] Naumann, U. *The Art of Differentiating Computer Programs*. Software, Environments, and Tools (Society for Industrial and Applied Mathematics, 2011). URL https://doi.org/10.1137/1.9781611972078.

[15] Arruda, J., Bracher, N., Köthe, U., Hasenauer, J. & Radev, S. T. Diffusion models in simulation-based inference: A tutorial review. *arXiv* (2025). URL https://doi.org/10.48550/arXiv.2512.20685.

[16] Radev, S. T. *et al.* BayesFlow: Amortized Bayesian workflows with neural networks. *Journal of Open Source Software* **8**, 5702 (2023). URL https://doi.org/10.21105/joss.05702.

[17] Schälte, Y., Klinger, E., Alamoudi, E. & Hasenauer, J. pyABC: Efficient and robust easy-to-use approximate Bayesian computation. *Journal of Open Source Software* **7**, 4304 (2022). URL https://doi.org/10.21105/joss.04304.

[18] Andrieu, C., Doucet, A. & Holenstein, R. Particle Markov chain Monte Carlo methods. *Journal of the Royal Statistical Society: Series B (Statistical Methodology)* **72**, 269–342 (2010). URL https://doi.org/10.1111/j.1467-9868.2009.00736.x.

[19] Chopin, N. & Papaspiliopoulos, O. *An Introduction to sequential Monte Carlo*. Springer Series in Statistics (Springer International Publishing, 2020). URL https://doi.org/10.1007/978-3-030-47845-2.

[20] Andrieu, C. & Roberts, G. O. The pseudo-marginal approach for efficient Monte Carlo computations. *The Annals of Statistics* **37** (2009). URL https://doi.org/10.1214/07-AOS574.

[21] Wieland, V., Waßmuth, N., Contento, L., Kühn, M. & Hasenauer, J. Assessment of simulation-based inference methods for stochastic compartmental models in epidemiological research. *PLOS ONE* **21**, 1–28 (2026). URL https://doi.org/10.1371/journal.pone.0353306.

[22] Edlund, S. *et al.* Comparing three basic models for seasonal influenza. *Epidemics* **3**, 135–142 (2011). URL https://doi.org/10.1016/j.epidem.2011.04.002.

[23] Buchholz, U. *et al.* GrippeWeb - Daten des Wochenberichts [Data set].
[24] Legrand, J., Grais, R. F., Boelle, P.-Y., Valleron, A.-J. & Flahault, A. Understanding the dynamics of Ebola epidemics. *Epidemiology & Infection* **135**, 610–621 (2007). URL https://doi.org/10.1017/s0950268806007217.
[25] Rico, A. *et al.* Epidemiology of epidemic Ebola virus disease in Conakry and surrounding prefectures, Guinea, 2014–2015. *Emerging infectious diseases* **22**, 178 (2016). URL https://doi.org/10.3201/eid2202.151304.
[26] Plotdigitizer: Version 3.1.6 (2026). URL https://plotdigitizer.com.
[27] Kühn, M. J. *et al.* Assessment of effective mitigation and prediction of the spread of SARS-CoV-2 in Germany using demographic information and spatial resolution. *Mathematical Biosciences* 108648 (2021). URL https://doi.org/10.1016/j.mbs.2021.108648.
[28] Radev, S. T., Mertens, U. K., Voss, A., Ardizzone, L. & Köthe, U. BayesFlow: Learning complex stochastic models with invertible neural networks. *IEEE transactions on neural networks and learning systems* **33**, 1452–1466 (2020). URL https://doi.org/10.1109/TNNLS.2020.3042395.
[29] BMAS. Pendlerverflechtungen der sozialversicherungspflichtig Beschäftigten nach Kreisen - Deutschland (Jahreszahlen) (2022). URL https://statistik.arbeitsagentur.de/SiteGlobals/Forms/Suche/Einzelheftsuche_Formular.html?topic_f=beschaeftigung-sozbe-krpendd. Publication Title: www.bmas.de.
[30] Beneduce, C., Muñoz-Repiso, T. G., Lepri, B. & Luca, M. pyspainmobility: a python package to access and manage spanish open mobility data (2025). URL https://doi.org/10.48550/arXiv.2506.13385.
[31] Robert Koch-Institut. Intensivkapazitäten und COVID-19-Intensivbettenbelegung in Deutschland (2025). URL https://doi.org/10.5281/zenodo.17046865.
[32] Robert Koch-Institut. SARS-CoV-2 Infektionen in Deutschland (2025). URL https://doi.org/10.5281/zenodo.17046972.
[33] Ministerio de Sanidad. Datos abiertos de capacidad asistencial (2023). URL https://www.sanidad.gob.es/areas/alertasEmergenciasSanitarias/alertasActuales/nCov/documentos/Datos_Capacidad_Asistencial_Historico_14072023.csv.
[34] Ministerio de Sanidad. Evolución de enfermedad por el coronavirus (covid-19) (2023). URL https://datos.gob.es/en/catalogo/e05070101-evolucion-de-enfermedad-por-el-coronavirus-covid-19.
[35] Prem, K., Cook, A. R. & Jit, M. Projecting social contact matrices in 152 countries using contact surveys and demographic data. *PLoS computational biology* **13**, e1005697 (2017). URL https://doi.org/10.1371/journal.pcbi.1005697.
[36] Ward, D., Cannon, P., Beaumont, M., Fasiolo, M. & Schmon, S. Robust neural posterior estimation and statistical model criticism. In Koyejo, S. *et al.* (eds.) *Advances in Neural Information Processing Systems*, vol. 35, 33845–33859 (Curran Associates, Inc., 2022). URL https://proceedings.neurips.cc/paper_files/paper/2022/file/db0eac6747e3631eb91095cd76065611-Paper-Conference.pdf.
[37] Kerr, C. C. *et al.* Covasim: An agent-based model of COVID-19 dynamics and interventions. *PLOS Computational Biology* **17**, 1–32 (2021). URL https://doi.org/10.1371/journal.pcbi.1009149. Model repository: https://github.com/InstituteforDiseaseModeling/covasim.
[38] Fumanelli, L., Ajelli, M., Manfredi, P., Vespignani, A. & Merler, S. Inferring the Structure of Social Contacts from Demographic Data in the Analysis of Infectious Diseases Spread. *PLoS Computational Biology* **8**, e1002673 (2012). URL https://doi.org/10.1371/journal.pcbi.1002673.
[39] Schmidt, A., Zunker, H., Heinlein, A. & Kühn, M. J. Graph neural network surrogates to leverage mechanistic expert knowledge towards reliable and immediate pandemic response. *Scientific Reports* **16** (2026). URL https://doi.org/10.1038/s41598-026-39431-5.
[40] Abadi, M. *et al.* TensorFlow: Large-scale machine learning on heterogeneous systems (2015). URL https://www.tensorflow.org/. Software available from tensorflow.org.
[41] Grattarola, D. & Alippi, C. Graph Neural Networks in TensorFlow and Keras with Spektral [Application Notes]. *IEEE Computational Intelligence Magazine* **16**, 99–106 (2021). URL https://doi.org/10.1109/MCI.2020.3039072.
[42] Bianchi, F. M., Grattarola, D., Livi, L. & Alippi, C. Graph Neural Networks with Convolutional ARMA Filters. *IEEE Transactions on Pattern Analysis and Machine Intelligence* **PP**, 1–1 (2021). URL https://doi.org/10.1109/TPAMI.2021.3054830.
[43] BMAS. Pendlerverflechtungen der sozialversicherungspflichtig Beschäftigten nach Kreisen - Deutschland (Jahreszahlen) (2020). URL https://statistik.arbeitsagentur.de/SiteGlobals/Forms/Suche/Einzelheftsuche_Formular.html?topic_f=beschaeftigung-sozbe-krpendd. Publication Title: www.bmas.de.
[44] Jones, T. C. *et al.* Estimating infectiousness throughout SARS-CoV-2 infection course. *Science* **373**, eabi5273 (2021). URL https://doi.org/10.1126/science.abi5273.

[45] Ke, R. *et al.* Daily longitudinal sampling of SARS-CoV-2 infection reveals substantial heterogeneity in infectiousness. *Nature Microbiology* **7**, 640–652 (2022). URL https://10.1038/s41564-022-01105-z.

[46] DESTATIS Zeitverwendungserhebung (ZVE) 2022. Available online: https://www.destatis.de/DE/Themen/Gesellschaft-Umwelt/Einkommen-Konsum-Lebensbedingungen/Zeitverwendung/Tabellen/aktivitaeten-insgesamt-zve.html, accessed 2025-08-22.

[47] Nyberg, T. *et al.* Risk of hospital admission for patients with SARS-CoV-2 variant b.1.1.7: cohort analysis. *BMJ* **373**, n1412 (2021). URL https://doi.org/10.1136/bmj.n1412.

[48] Zali, A. *et al.* Mortality among hospitalized COVID-19 patients during surges of SARS-CoV-2 alpha (b.1.1.7) and delta (b.1.617.2) variants. *Scientific Reports* **12**, 18918 (2022). URL https://doi.org/10.1038/s41598-022-23312-8.

[49] Jülich Supercomputing Centre. JURECA: Data Centric and Booster Modules implementing the Modular Supercomputing Architecture at Jülich Supercomputing Centre. *Journal of large-scale research facilities* **7**, A182 (2021). URL https://doi.org/10.17815/jlsrf-7-182.

[50] DESTATIS Bevölkerung: Deutschland, Stichtag, Altersjahre. Available online: https://www-genesis.destatis.de/datenbank/online/table/12411-0005/search/s/MTI0MTEtMDAwNQ==, accessed 2025-08-22.

[51] DESTATIS Erwerbstätigenquoten 1991 bis 2024. Available online: https://www.destatis.de/DE/Themen/Arbeit/Arbeitsmarkt/Erwerbstaetigkeit/Tabellen/erwerbstaetigenquoten-gebietsstand-geschlecht-altergruppe-mikrozensus.html, accessed 2025-08-25.

[52] DESTATIS Vorausberechnete Privathaushalte: Deutschland, Jahre, Varianten der Haushaltsvorausberechnung, Haushaltsgröße. Available online: https://www-genesis.destatis.de/datenbank/online/table/12421-0100/search/s/MTI0MjEtMDEwMA==, accessed 2025-08-25.

[53] DESTATIS Schüler: Deutschland, Schuljahr, Geschlecht, Schulart, Jahrgangsstufen. Available online: https://www-genesis.destatis.de/datenbank/online/table/21111-0002/search/s/MjExMTEtMDAwMg==, accessed 2025-08-25.
